# The Interplay between Immunity and Microbiota at Intestinal Immunological Niche: The Case of Cancer

**DOI:** 10.3390/ijms20030501

**Published:** 2019-01-24

**Authors:** Rossella Cianci, Laura Franza, Giovanni Schinzari, Ernesto Rossi, Gianluca Ianiro, Giampaolo Tortora, Antonio Gasbarrini, Giovanni Gambassi, Giovanni Cammarota

**Affiliations:** 1 Department of Internal Medicine, Università Cattolica del Sacro Cuore, Fondazione Policlinico Universitario A. Gemelli IRCCS, Largo A. Gemelli, 8, 00168 Roma, Italy; laura.franza01@icatt.it (L.F.); giovanni.gambassi@unicatt.it (G.G.); 2 Department of Medical Oncology, Università Cattolica del Sacro Cuore, Fondazione Policlinico Universitario A. Gemelli IRCCS, Largo A. Gemelli, 8, 00168 Roma, Italy, giovanni.schinzari@unicatt.it (G.S.); ernestorossi.rm@gmail.com (E.R.); giampaolo.tortora@policlinicogemelli.it (G.T.); 3 Department of Gastroenterology, Università Cattolica del Sacro Cuore, Fondazione Policlinico Universitario A. Gemelli IRCCS, Largo A. Gemelli, 8, 00168 Roma, Italy; gianluca.ianiro@hotmail.it (G.I.); antonio.gasbarrini@unicatt.it (A.G.); giovanni.cammarota@unicatt.it (G.C.)

**Keywords:** gut microbiota, immunological niche, dysbiosis, cancer, immune system

## Abstract

The gut microbiota is central to the pathogenesis of several inflammatory and autoimmune diseases. While multiple mechanisms are involved, the immune system clearly plays a special role. Indeed, the breakdown of the physiological balance in gut microbial composition leads to dysbiosis, which is then able to enhance inflammation and to influence gene expression. At the same time, there is an intense cross-talk between the microbiota and the immunological niche in the intestinal mucosa. These interactions may pave the way to the development, growth and spreading of cancer, especially in the gastro-intestinal system. Here, we review the changes in microbiota composition, how they relate to the immunological imbalance, influencing the onset of different types of cancer and the impact of these mechanisms on the efficacy of traditional and upcoming cancer treatments.

## 1. Introduction

Mounting evidence has conclusively established that the gut microbiota is involved in the pathogenesis of several medical conditions, such as inflammatory [1,2], liver [3,4], pancreatic [5], and pulmonary diseases [6], neurological [7] and skin disorders [8], and cancer [9,10,11].

Gut microbiota comprises all of the microorganisms residing in the human intestine, including bacteria, viruses, fungi, archea and protozoa. It contains more than 1000 different bacterial species, over 100 times more than the total number of host cells [12].

Germ-free mice models have shown that the gut microbiota plays some pivotal functions in the development and modulation of several organs and systems, such as the immune and endocrine system, blood, liver and lungs [13]. In the intestine, gut microbiota is able to maintain epithelial homeostasis to support the development of gut associated lymphoid tissue (GALT). Microbiota also enhances epithelial cytokine production, which regulates the action of T and B lymphocytes, macrophages and polimorphs [14,15]. Cytokines, such as interleukin (IL)-1β, tumor necrosis factor (TNF)-α, IL-2, IL-6, IL-15, IL-21, IL-23, can determine an inflammatory response, while others, such as IL-10 and transforming growth factor (TGF)-β, have anti-inflammatory effect. The balance between these two classes is responsible for the overall inflamed or homeostatic status of the gut [16].

In a healthy state, there is a perfect balance between gut microbiota and immune system at gut interface [17]. The breakdown of this physiological balance in microbial composition precipitates a pathological state known as ‘gut dysbiosis’, contributing to the overgrowth of pathogen bacteria in the intestinal lumen. Dysbiosis is considered a common effector in the different pathogenetic pathways involved in several human diseases [18,19,20]. Many factors, such as age, hormonal perturbations, diet composition and supplement intake, antibiotic therapies, lifestyle and physical activity exert an impact on gut microbiome and equilibrium [21,22]. Dysbiosis can also be a consequence of an inflammatory status: In genetically susceptible patients, dietary compounds, toxins and antibiotics can start a low-grade inflammation, leading to dysbiosis. In patients suffering from IBD, for example, high calorie and high fat diets, typical of the western world, have been shown to determine a worsening of the inflammatory status of the gut [23].

There appears to be a bidirectional relationship between host immunity and gut microbiota. On one hand, the development of host immunity is mediated by microbiota but, on the other hand, the microbiota itself is constantly modulated by host immunity. This permanent cross-talk between mucosal immunity and gut microbiota is responsible, for example, for the anergy of host immune cells against its own antigens and dietary ones. In fact, microbiota-driven dendritic cells (DC), particularly the CD103+ subset, can induce expression of a subset of T cells with regulatory functions (T-regs) and their related anti-inflammatory cytokines. As well, B-regulatory cells (B-regs) take part in this process, suppressing effector T cells and contributing to the overall process of immune tolerance to food antigens [24].

Here, we review the complex interaction between immune system and microbiota at the gut ‘immunological niche’ interface and its role in development, growth and spreading of different types of gastro-intestinal cancers.

## 2. Immune System and Cancer

**Figure 1:**
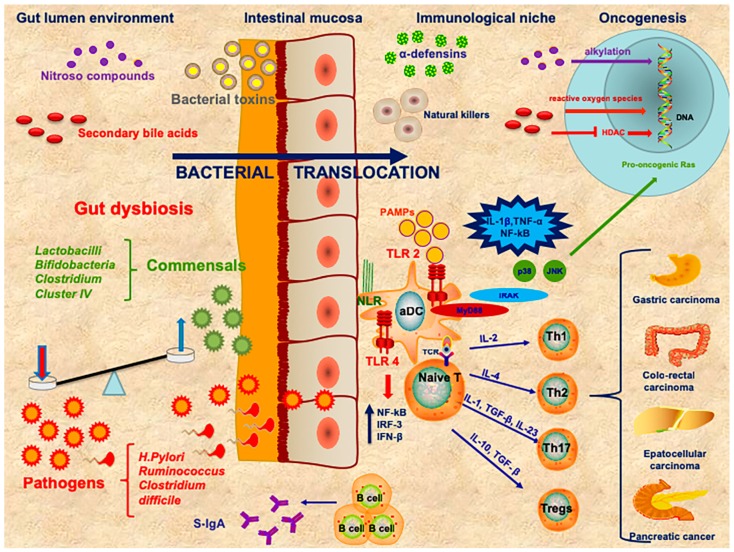
The complex interplay among gut lumen environment, mucosal barrier, immunological niche in oncogenesis. The failure of maintaining homeostatic equilibrium between commensals and pathogens at gut lumen level leads to dysbiosis. The bacterial products enhance the gut permeability leading to bacterial and toxins translocation. Toll-like receptors (TLRs) expressed on activated dendritic cells (aDC) are able to recognize pathogen-associated molecular patterns (PAMPs) and can activate the NF-κB, JNK and p38 mitogen-activated protein kinases. JNK promotes the activation of pro-oncogenic *Ras*. Other receptors situated on several types of immune cells are represented by nucleotide-binding oligomerization domain-like receptors (NLRs), which are pattern recognition receptors (PRRs) that can activate NF-κB and promote inflammasomes. Other carcinogenetic agents, like nitrous compounds and secondary bile acids, can act respectively as alkylating mediators or via reactive oxygen species at a DNA level. Furthermore, high doses of butyrate inhibit histone deacetylase (HDAC) that is able to inactivate many oncogenic signaling pathways. The presence of pro-inflammatory T-cells can induce pro-inflammatory cytokines at tumor site. The concomitant action of T-regs creates a state of immunosuppression at tumor level.

Cancer and the immune system are inextricably linked. A similar strong interaction between gut microbiota and innate and adaptive immunity has also been established. A complex network of cytokines regulates the interplay between bacteria, viruses, parasites and fungi and mucosal immune cells [12] (Figure 1).

Toll like receptors (TLRs) are a component of innate immunity. They are germline-encoded type I transmembrane receptors, expressed on epithelial cells (e.g., intestinal cells) and on various immune system-related cells (e.g., T-lymphocytes, macrophages and dendritic cells, DCs). TLRs serve as pathogen recognition receptors (PRRs) and recognize pathogen-associated molecular patterns (PAMPs) that are specific and essential for microbes [25]. Among the different TLRs, TLR3 and TLR4 are able to activate both the transcription nuclear factor kappa-light-chain-enhancer of activated B cells (NF-κB) and the interferon regulatory factor 3 (IRF3) that induces interferon-beta (IFN-beta) production [26]. Many others TLRs also lead to the activation of mitogen-activated protein (MAP) kinases p38. This, in turn, increases the expression of many pro-inflammatory genes, via adaptor molecules, such as Myeloid differentiation primary response gene 88 (MyD88), which is able to recruit IRAKs (IL-1R-associated kinase family). The activation of MAP3 kinases follows and determines the activation of NF-κB, c-Jun N-terminal kinase (JNK) and MAP kinases p38.

Studies on MyD88-deficient mice have documented that TLRs’ response to PAMPs of commensal bacteria plays a fundamental role in epithelial cell homeostasis [27], induction of antimicrobial peptides [28,29], and in the modulation of the adaptive immune response [30,31]. In contrast, bacteria-activated TLRs may mediate inflammation and carcinogenesis. Indeed, cancer cells present high expression of TLRs [32], while, MyD88-deficient mice are less prone to develop tumors [33]. In this respect, several recent studies [34,35] have pointed towards a tumor promoting function, due to the activation of pro-oncogenic Ras by JNK signaling. This inhibits apoptosis and enhances expression of metallo-proteinases [36].

In the 1800s, Virchow described for the first time a large number of lymphocytes (lymphocytes infiltrating tumor or TILs), present at the tumor site [37]. Based on this observation, he hypothesized a role of the immune system in cancer development, growth and spreading.

Only many years later, thanks to technological advances, it was possible to isolate TILs and CD8+ cytotoxic T-lymphocytes (CTLs) from peripheral blood in neoplastic patients. CD8+ CTLs play a pivotal role against cancer because they are able to kill malignant cells upon recognition by T-cell receptor (TCR) of specific antigenic peptides present on the surface of target cells [16]. The existence of a tumor-specific CTLs response was further supported by the identification of tumor-associated antigens (TAA) and by the detection of TAA-specific CD8+ T-cells in spontaneously regressing tumors. Moreover, it has been recently demonstrated that, in colorectal cancer, TILs are predominantly CD4+ T cells and produce pro-inflammatory cytokines, such as IFNγ and IL-17. On the other hand, there is also a subset of CD4+ cells producing IL-4, which appear to favor Th2 phenotype, which seems to favor oncogenesis [38]. Another subset of immune cells presents itself at tumor a site in longer surviving neoplastic patients and is represented by natural killers (NK). These cells are able to trigger tumor apoptosis and inhibit cell proliferation [39].

On the other hand, many studies have shown that at the site of the tumor there is an overall immunosuppressed state. Such condition is obtained by cancer cells themselves through the production of immunosuppressive factors (e.g., TGF-β) and/or by recruiting regulatory immune cells with immunosuppressive functions (e.g., T regulatory cells, T-regs). The prevalence of T-regs and the prognosis of tumors are inversely correlated [40]. T-regs modulate tumor-specific effector T-lymphocytes by producing immunosuppressive cytokines, such as IL-10 and TGF-β, consuming IL-2 or expressing the inhibitory molecule cytotoxic T-lymphocyte associated protein 4 (CTLA-4 or CD 152). T-regs can also inhibit the proliferation of pro-inflammatory subsets of CD4+-T lymphocytes (T-helper or Th) and stimulate B lymphocytes to produce specific immunoglobulins. Th17 and signal transducer and activator of transcription 3 (STAT3) have been implicated in carcinogenesis of various human systems [41,42] by increasing cell proliferation and inhibiting apoptosis [16,43,44]. Th17 produces pro-inflammatory cytokines, such as IL-17 and IL-23 that promote tumor growth [45]. Moreover, Th17 can induce production of Th1-related pro-inflammatory cytokines, chemokine (C-X-C motif) ligand 9 and 10 (CXCL9 and CXCL10), at the tumor site. Th17 cells have similar characteristics to stem cells and are able to renew themselves and, at the same time, they can stimulate the production of Th1-like effectors. The cytokinic environment present at the tumor site influences the different patterns of expression of Th17 cells: In colorectal, hepatocellular and pancreatic cancers, Th17 is associated to a worse prognosis, as it favors immune tolerance towards the tumor, while in ovarian cancer it improves patients’ life expectancy [40,46]. In cancer patients, T cells, persistently stimulated by tumor antigens, tend to lose their ability to express cytokines or attack target cells. This phenomenon is known as T-cell exhaustion and is probably the most common mechanism of immune escape [47]. When such condition ensues, the tumor is able to continue growing regardless of the initial immune response [48,49].

## 3. The Role of Gut Microbiota in Cancer

A growing body of evidence supports the notion that gut microbiota is able to interfere both with cancer development and with response to anti-cancer therapies (Table 1).

Gut microbiota can generate signaling molecules and microbial products, which are potentially toxic for the intestinal mucosal surface [15]. These products increase gut permeability to foreign antigens [5], and a leaky gut facilitates carcinogenesis, mainly, by enhancing inflammation and by influencing gene expression [32]. There is evidence, for example, that the quantity and quality of gut microbial species changes in genetically-predisposed individuals and/or in individuals affected by pre-neoplastic inflammatory disorders [50]. Furthermore, a gut dysbiosis has been documented in association to several tumors. On the other hand, germ-free animal models display a noteworthy reduced cancer incidence and this seems related to the absence of gut dysbiosis and mucosal inflammation [51].

Another important mechanism through which microbiota exerts an anti-neoplastic action is through dietary fibers. Dietary fibers are not metabolized and represent the substrate of saccharolytic fermentation with production of short/chain fatty acids (SCFAs), such as butyrate, propionate and acetate. SCFAs are able to suppress inflammation and expression of pro-carcinogenics and to downregulate tumor growth [52,53]. Lactobacilli and bifidobacteria maintain homeostasis in the gastrointestinal tract [54] and are the principal actors in the fiber fermentation process [55].

Yet, SCFAs are able to bind other bacterial metabolites, like secondary bile acids, that can promote and/or enhance the inflammation, oxidative DNA damage and subsequent carcinogenesis [56] and cancer growth. The different effects of butyrate are determined by its concentration. When present in large quantities, it is able to inhibit cancer cell proliferation, independently from the Warburg effect, through inhibition of histone deacetylase (HDAC), that is able to inactivate many oncogenic signaling pathways [15] and lower doses of butyrate are, instead, capable of inducing histone acetylation and not act as a HDAC inhibitor. Humphreys et al. [57] have demonstrated that butyrate supplementation reduces the level of pro-oncogenic miRNA, such as miR-17-92, in rectal biopsies. Moreover, it promotes the expression of TLR4, MAPK and NF-κB phosphorylation [58]. Butyrate is also linked to the capability to promote the T-regs proliferation and has an immune-modulating role [59,60], overall leading to some controversy on its effect [15]. Other data suggest that colonic cell response to SCFAs may be determined by the expression of caspase and peroxisome proliferator-activated receptor γ (PPARγ), implying that interactions between gut microbiota and the host are heavily influenced by the individual’s genetics [61].

Microbiota and host genetics undergo a complex cross-talk, which determines for example that patients with a genetic predisposition may more easily face dysbiosis and have fewer SCFAs-producing bacteria [62].

Gut microbiota composition varies largely with age, lifestyle and lifelong dietary intake but it also modified by medications, especially antimicrobials [63]. The relation between use of antibiotics and development of cancer remains quite controversial. In fact, in an experimental murine model, antibiotics have been shown to arrest tumor progression [32]. On the contrary, recent data lends support to the hypothesis that repeated antibiotic use leads to alteration in microbiota composition, with subsequent pro-carcinogenetic modifications [64] in the gut, mostly pancreas and intestine, but also elsewhere. Penicillin use, in particular, appears to be a risk factor for the insurgence of esophagus, stomach and pancreas malignancies [65].

## 4. Esophageal and Stomach Cancer

The microbiota of the esophagus is more similar to the oral microbiota than to the intestinal one. In physiological conditions, the esophageal microbial population is characterized by *Firmicutes*, *Bacteroides*, *Actinobacteria*, *Proteobacteria*, *Fusobacteria* and *TM7* and is dominated by the genus *Streptococcus*. Instead, in patients with gastro-esophageal reflux and Barrett esophagus, for example, there is a higher presence of *Bacteroides*, *Proteobacteria* and *Fusobacterium*, and an overall increased diversity, finally resembling more the stomach microbiome [66].

*Helicobacter pylori* (*Hp*) is considered a class 1 human carcinogen for gastric adenocarcinoma [67]. In gastric samples and in the serum of mice with *Hp* associated gastric cancer, there are increased levels of IL-1, IL-17 and TNF-α, highlighting an enhanced Th17 response [40]. *Hp* has also been associated to low grade gastric mucosa associated lymphoid tissue (MALT) lymphoma and it seems that treating *Hp* in patients with a MALT lymphoma can determine a remission of the lymphoma itself [68,69]. Bacterial overgrowth is typically present in gastric tumors not *Hp*-related [70]. In these patients the continuous cross-talk between different species, particularly *Pasteurella stomatis*, *Dialister pneumosintes*, *Slakia exigua*, *Parvimonas micra* and *Streptococcus anginosus*, probably plays a key role in disease progression [71]. Surprisingly, *Hp* exerts a protective action in esophageal cancer [69]. Although not conclusively explained, this protection could be due to the reduced gastric acid secretion it induces [72].

In general, patients suffering from esophageal and gastric cancer present higher amount of T-regs compared to healthy subjects, especially among patients at advanced stage of disease or with the worst prognosis [73,74]. A recent study has shown that Enterobacteriaceae, in particular *Ruminococcus*, are significantly higher in patients with stomach cancer [56], and it could represent the initial trigger for the altered immunologic status in these patients.

## 5. Colorectal Cancer

Chen et al. have reported that an imbalance in gut microbiota composition is associated with colorectal cancer [75].

For example, Lactobacillaceae decrease in number in colon cancer patients, while they increase after anti-neoplastic treatment [56]. Indeed, *Lactobacilli* have been shown to block the growth of colon carcinoma [76]. Bifidobacteriaceae are also reduced in patients with rectal tumor and this could lead to a reduced folate synthesis, possibly favoring chromosomal instability. In addition, *Bifidobacterium* exerts a competitive action against pathogens and regulate immune system cells [77].

The pathogens that appear to be primarily involved in the pathogenesis of colorectal cancer [78] are *Streptococcus bovis* (*S. bovis*) [79], *Hp* [80], *Bacteroides fragilis* (*B. fragilis*) [81], *Enterococcus faecalis* (*E. faecalis*) [82], *Clostridium septicum* (*C. septicum*) [83], *Fusobacterium* spp. [84] and *Escherichia coli* (*E. coli*) [85]. Some of these bacteria have a direct carcinogenic effect. This is true for *Hp* or for some strains of *Escherichia coli* that produce colibactin, a genotoxin implicated in the onset of colorectal cancer [86]. Other microbial species act in more subtle ways. Enterotoxigenic *B. fragilis*, for example, appears to play a role in the development of colorectal carcinoma through immune-modulation via Th17. On the other hand, *B. fragilis* can determine metaplasia through the STAT-3 pathway and the strain that produces the *B. fragilis* toxin (BFT) activates the WNT and NF-κB signaling pathways, leading to a chronic inflammatory status [87,88]. *S. bovis* increases the tumors capacity of immunologic escape but it also creates a symbiotic relationship with neoplastic cells, favoring their growth [89]. The role of *E. faecalis* in cancerogenesis is ambiguous: On the one hand it reportedly increases in patients with colorectal cancer [90] and causes an inflammatory status that benefits the tumor through production of ROS, which has a damaging effect on the DNA [91]. On the other hand, it has recently been suggested that the association between colorectal cancer and *E. faecalis* is prevalently due to an altered intestinal environment in patients with colorectal cancer. In this scenario, *E. faecalis* may benefit from an already compromised situation, which allows it to grow undisturbed and uncontrolled, determining an increased virulence, which can further damage the epithelial tissue [92].

Overall, gut dysbiosis acts as a colorectal cancer promoter through a series of mechanisms, which involve immune-modulation, toxins production, metabolic activities and increased oxidative stress and inflammation in the intestinal environment [78].

## 6. Hepatocellular Carcinoma

The liver does not have its own microbiome and is influenced by gut microbiota metabolites through the entero-hepatic circulation [93].

Although it cannot be formally described as liver microbiota, there are microbial species capable to colonize it, most specifically hepatotropic viruses, such as *hepatitis B virus* (*HBV*) and *hepatitis C virus* (*HCV*). Such viruses increase considerably the risk of developing hepatocellular carcinoma [94]. At least part of this increased risk is explained by a direct action on liver cells through epigenetic mechanisms. *HBV* modifies methylation on p16 (INK4A), glutathione S-transferase P 1 (GSTP1), CDH1 (E-cadherin), *Ras association domain containing protein 1* (RASSF1A), *p21* (WAF1/CIP1) genes, while *HCV* alters methylation on suppressor of cytokine signaling 1 (SOCS-1), growth arrest and damage inducible beta (Gadd45β), O^6^-alkylguaniline DNA alkyltransferase (MGMT), STAT1 and antigen presenting cells (APC). As well, effects on histone proteins, chromatin, and noncoding RNAs have been described [95]. In addition, *HCV* is a well-known immune-modulator; in murine models, for example, it increases FAS-mediated apoptosis of T lymphocytes [96]. At the same time, both *HCV* and *HBV* appear to determine gut dysbiosis, that contributes to disease progression [97].

Hepatocellular carcinoma is often a late evolution of a chronic liver disease. Certain gut microbial species seem to either facilitate or slow down such process [98,99]. Bacteria belonging to the *Helicobacter spp* (*pylori* and *hepaticus*, in particular) have been linked to an increased risk of liver cancer. There appear to be various mechanisms through which *H. hepaticus* is able to determine a carcinogenic effect. Not only it can directly damage DNA, activating the WNT and NF-κB signaling pathways in tumor cells, but it also appears to be able to suppress intra-tumor immunity in aflatoxin- and *hepatitis C virus*-induced HCC [100,101]. *Escherichia coli* has also been linked to the development of hepatocellular carcinoma; cirrhotic patients who developed a hepatocellular carcinoma have a microbiome enriched with *E. coli*, when compared to those who did not develop the tumor [102].

It is noteworthy that a leaky gut increases the number of toxins and bacteria potentially reaching the liver. The related state of chronic inflammation can promote non-alcoholic liver disease and fibrosis and could trigger the development of tumors [103]. For example, in obese patients the microbiota is characterized by an increase in *Firmicutes/Bacteroidetes* ratio and by an overall reduction of the number of bacterial species [104,105]. This dysbiosis favors fat storage, leading to a fatty liver and a metabolic syndrome, both established risk factors for hepatocellular carcinoma [106,107].

One of the most studied risk factors for hepatocellular carcinoma is alcohol consumption. Alcohol has a direct toxicity on the liver, but it also has important effects on gut microbiome [108]. Some studies even suggest that restoring and maintaining a normal eubiosis is able to, at least, slow down the progression of alcohol-related liver disease [109]. Yet, evidence is still scarce and further investigations are necessary. On the other hand, *Lactobacillus* species, *Bifidobacterium* species, *Parabacteroides* species, and *Oscillibacter* species, appear to have a protective effect on the liver, through their immune-modulating properties [110].

## 7. Pancreatic Cancer

Pancreatic adenocarcinoma remains one of the most lethal tumors overall. Several reports have proposed a pathogenetic role of *Helicobacter pylori* in pancreatic cancer [111]. *Helicobacter* seems to activate the NF-κB pathway and its lipopolysaccharide triggers *KRAS* gene mutation, which is present in 90% of pancreatic adenocarcinomas [112,113,114]. As well, *Hp* may enhance the activator of signal transducer and activator of transcription3 (STAT3) implicated in carcinogenesis through its capacity to promote cellular proliferation and, conversely, inhibit apoptosis [115,116]. Despite this supportive evidence, a recent meta-analysis, based on prospective epidemiologic studies, has not documented a strong association between *Hp* infection and pancreatic cancer [117].

As for the liver, the pancreas does not have its own microbiota. As such, it is foreseeable that the pancreas is influenced by the gut and oral microbiota [118].

In colon samples of patients with pancreatic carcinoma, for example, Youssef et al. have found reduced levels of *Lactobacilli* and *Parabacteroides* [56]. These species have a proven anticancer function, as they reduce TLR4 signaling pathway [119]. Moreover, levels of *Lactobacilli* are restored after anticancer treatment. Another study has linked pancreatic adenocarcinomas to decreased gut microbiota diversity, caused by an increase of LPS-producing bacteria and a decrease of both alpha diversity and butyrate-producing bacteria [63].

Geller et al. found increased levels of Enterobacteriaceae, Pseudomonadaceae, Moraxellaceae and Enterococcaceae in pancreatic cancer tissue [120]. Furthermore, Mei et al. studying the duodenal microbiota of patients with pancreatic cancer identified mostly *Acinetobacter, Aquabacterium*, *Oceanobacillus*, *Rahnella*, *Massilia*, *Delftia*, *Deinococcus*, and *Sphingobium*, while healthy controls harbored *Porphyromonas, Escherichia, Shigella* and *Pseudomonas* [111].

More recently, pancreatic cancer has been associated to a particular salivary microbiota. The presence of periodontal pathogens, such as *Porphyromonas gingivalis* (strain ATCC 53978) has been associated with an increased risk of pancreatic cancer, while the opposite is true for the presence of *Neisseria elongate* and *Streptococcus mitis* [121]. Furthermore, Gammaproteobacteria have been linked to pancreatic cancer and when transferred to mice, these bacteria induced gemcitabine resistance [122].

## 8. The Role of Microbiota in Cancer Therapy

The ability of gut microbiota to modulate the response to cancer chemotherapy and immunotherapy has been first observed in mice [123]. Recently, evidence has emerged revealing that certain clusters of gut microbiota may be related to chemotherapy outcome in several human epithelial solid tumors, such as lung and renal carcinomas, and melanoma [123]. The effects of microbiota on cancer treatment are unlikely due to a single specie but rather to changes in the ecology and metabolism of gut microbiota impacting cancer immunity altogether [124].

Patients who undergo chemotherapy have a higher risk of developing a leaky gut as a direct consequence of chemotherapy itself [125]. Leaky gut and dysbiosis appear to decrease the efficacy of platinum compounds [126]. As well, the effect of other anti-neoplastic agents is modified by gut microbiota composition. *Mycoplasma hyorhinis* and cytidine-deaminase-positive Proteobacteria are able to metabolize and modify gemcitabine, impairing its anti-tumor action and such effect is reversed with antibiotic therapy [120,127]. Likewise, the action of cyclophosfamide is influenced by gut microbiota composition. Bacterial translocation creates an inflamed environment that promotes IFN-γ-producing γδ-T-cells migration in the tumor area [128].

Microbiota appears to also modulate the response to radiotherapy as germ free mice are less susceptible to the toxicity of radiation than conventionally raised mice [126]. Gut microbiota might influence the outcomes of cancer patients who are treated surgically with effects ranging from altered wound healing to permanent dysbiosis, to selection of resistant and virulent microbial species [129].

Anti-neoplastic immunotherapies have been successfully used in melanoma and aim at activating and expanding tumor-specific CTLs, with the goal of destroying primary cancer cells and metastases [130]. The most promising current cancer immunotherapies, utilized not only in melanoma but in several solid epithelial tumors, act on immune checkpoint molecules anti-programmed death 1 (PD-1) and anti-CTLA-4 immunotherapies. PD-1 is an immuno-inhibitory lymphocyte receptor involved in the maintenance of peripheral tolerance to self. The interaction of PD-1 with its ligands, above all PD-L1 (CD274), causes the inhibition of CD8+T cell proliferation, survival and effector functions, and induces the CD4+ to Foxp3 T-cell differentiation increasing immune tolerance. CTLA-4, on the other hand, binds to CD80 or CD86 expressed on the surface of T-lymphocytes, and it causes a state of anergy in these cells. Some tumors (e.g., melanoma, prostate, kidney, lung) have the capacity to stimulate the exhaustion and anergy pathways, which is the main cause of immunologic escape capacity of these malignancies [131]. The PD-1/PD-L1 and the CTLA-4/B7 blockade has been shown to at least partly reverse immune alterations that determine T-cells exhaustion and anergy [132].

Even though these therapies are extremely promising, not all patients respond and some even experience severe side effects [133]. One of the main suspects of the very high variability in patient response is gut microbiome [134]. Marinelli et al. [135] have suggested that different bacterial species are involved in patients’ response to immunotherapy. In this respect, germ free mice, for example, are not able to respond to CTLA-4 blockage [136].

Another aspect that needs to be considered is host genetics, which is an important element in determining whether the patient will respond or not to immunotherapy. Patients with a genetically determined T-cell impairment, for example, do not respond well to immunotherapies [137]. Polymorphysms of TLR4 are linked to different outcomes in patients with breast tumors, while other immune-related loci (e.g., TNF-α, NF-κB, Janus kinases (JAK)/STAT proteins, Fc receptors FcγRIII (CD16), nucleotide-binding oligomerization domain-containing protein 2 (NOD2), autophagy related protein 16 (ATG16) and inflammasome pathway proteins) have also been linked to differences in the response to immunotherapy against cancer. Overall, the immune status of the host proves to be the primary factor in determining the response to all anti-neoplastic therapies, both directly and also indirectly through alterations of the gut microbiota [138].

Immunotherapy can increase potentially dangerous bacterial species. Most specifically, it appears to increase the number of *Clostridiales*, and to decrease the number of *Bacteroidales* and *Burkholderiales,* which play a pivotal role in a correct response to therapy. Another central role played by gut microbiota is in the modulation of side effects from immunotherapy. For example, the presence of *Bacteroidetes phylum* appears to have a protective effect against checkpoint-blockade-induced colitis [139]. Overall, CTLA-4 blockage requires the presence of specific bacteria to work, while anti-PD-1 drugs appear to interact only partially with gut microbiota [140].

## 9. Conclusions

A healthy gut microbiota is fundamental in maintaining homeostasis in the immune system, which is also key in cancer development and response. Still, the full extent of the actions of gut microbiota is not yet completely understood. As we have reported in our review, there are both immune-modulated and direct effects it plays in carcinogenesis of the gastro intestinal tract, not only in districts such as the intestine, but also in the liver and the pancreas, which are not directly colonized by the various microbial species. While some microbial species promote a healthy gut and the correct development of the various components of the immune system, others are even capable of determining malignancies. The importance of gut microbiota has also been demonstrated in the response to therapy, as the metabolic pathways it favors or suppresses can severely affect patients’ outcomes. Many studies underline the importance of microbiota in modulating different drugs’ effects and, in some cases, being necessary for the chemotherapy agent to have any effect whatsoever. Therapeutic strategies such as surgery and radiotherapy are also influenced by the presence of a healthy gut microbiota.

Overall, modulating the gut microbiota could be beneficial not only for those patients who have cancer, but also as a preventive strategy in the general population. Gut microbiota is a key player in many different diseases and could be targeted specifically in each patient through a precision medicine approach, so to maximize individual benefit, choosing the best therapeutic strategy and taking into account host and tumor characteristics [141].

## Figures and Tables

**Table 1:** An overview on the most studied gut microbioma species involved in GI cancer.

	Site	Effect	Mechanism	References
***Neisseria elongate***	Oral	↓↓↓ pancreatic tumor	Promotes oral homeostasis.	[121]
***Streptococcus mitis***	Oral	↓↓↓ pancreatic tumor	Promotes oral homeostasis.	[121]
***Porphyromonas gingivalis* (strain ATCC 53978)**	Oral	↑↑↑ pancreatic tumor	Promotes oral dysbiosis and inflammation.	[121]
***Helicobacter pylori***	Stomach, liver, intestine	↑↑↑ gastric liver pancreatic colorectal tumor; ↓↓↓ esophageal tumor	Immune-modulating effect through Th17 pathway; promoting factor for dysbiosis; not clear protective properties in esophageal tumor.	[67,80,97,111,142]
***Helicobacter hepaticus***	Liver	↑↑↑ liver tumor	Directly damages DNA, through WNT and NF-κB signaling pathways in tumor cells; suppresses intra-tumor immunity in aflatoxin- and hepatitis C virus-induced HCC.	[97,100,101]
***Streptococcus bovis***	Intestine	↑↑↑ colorectal tumor	Immune-modulating effect; symbiotic relation with tumor cells.	[79,89]
***Bacteroidesfragilis***	Intestine	↑↑↑ progression colorectal tumor	Immune-modulating effect through TH17 pathway; promotion of WNT, NF-κB and STS-3 pathways; direct effect of BFT toxin.	[87,88,134,143]
***Enterococcus faecalis***	Intestine	↑↑↑ colorectal tumor	Inflammatory effect through ROS production; increases risk of epithelial damage	[82,92]
***Clostridium septicum***	Intestine	↑↑↑ colorectal tumor	Inflammatory effect; increases risk of infectious complications.	[83]
***Fusobacterium spp.***	Intestine	↓↓↓ colorectal tumor; ↑↑↑ esophageal tumor.	Immune-modulating effect. Esophageal dysbiosis marker.	[66,84,144]
***Escherichia coli***	Intestine, pancreas	↑↑↑ colorectal and liver tumor; ↓↓ pancreatic tumor	Direct epithelial invasion; production of nitrous compounds through eme-metabolism; promotes dysbiosis.	[85,86,102,145]
***Lactobacillum spp.***	Gastro intestinal apparatus	↓↓↓↓ malignancies	Promotes gut homeostasis; anti-inflammatory effects.	[54,55,56,76,110]
***Bifidobacter spp.***	Gastro intestinal apparatus	↓↓↓↓ malignancies; ↓↓ immunotherapy side-effects	Promotes gut homeostasis through competition with pathogens; anti-inflammatory effects.	[54,55,77,110]
***Clostridium cluster IV***	Gastro intestinal apparatus	↓↓↓↓ malignancies	Promotes gut homeostasis; anti-inflammatory effects.	[55]
